# Arts as an ecological method to enhance quality of work experience of healthcare staff: a phenomenological-hermeneutic study

**DOI:** 10.1080/17482631.2017.1333898

**Published:** 2017-06-13

**Authors:** Eva Bojner Horwitz, Christina Grape Viding, Elisabeth Rydwik, Ephrat Huss

**Affiliations:** ^a^ Department of Public Health and Caring Sciences, Uppsala University, Uppsala, Sweden; ^b^ Center for Social Sustainability, Department of Neurobiology, Care Sciences and Society, Department of Clinical Neuroscience Karolinska Institutet, Stockholm, Sweden; ^c^ Department of Neurobiology, Care Sciences and Society, Division of physiotherapy, Karolinska Institutet, Solna, Sweden; ^d^ Stockholm County Council, Academic Primary Care Center, CEFAM, Sweden; ^e^ Charlotte B and Jack J. Spitzer Department of Social Work, Ben-Gurion University of the Negev, Beer-Sheva, Israel

**Keywords:** Arts-based activities, focus groups, healthcare worker, humanizing work-climate, phenomenological-hermeneutic

## Abstract

This paper explores the impact of self-chosen arts-based recreational activities, as opposed to the traditional arts therapy activities, on the well-being of healthcare providers. Three qualitative case studies of programs in which arts-based activities were used to work with healthcare providers, lasting for 10 weeks each, are phenomenological-hermeneutically evaluated using interviews and focus groups. The findings show what we refer to as an “ecological” ripple of effects: (1) the arts-based activities helped to reduce individual stress and to enhance mood over time, (2) the activities helped to transform workplace relationships within wards, and (3) the arts humanized the overall work climate in the healthcare setting. These effects go beyond those of using the art production as a strategy for stress reduction and imply potential for a more encompassing role for the arts within healthcare.

## Introduction

The effects of burnout, compassion, fatigue, overwork, and secondary trauma on healthcare staff are well documented (Grossi, Perski, Osika, & Savic, 2015; Swedish Social Insurance Agency, 2015). Indeed, healthcare staff mental stress is documented not only as a major issue for the individual practitioner but also for the organization and for the quality of care received by patients (Asberg et al., 2010; Bourbonnais, Brisson, & Vézina, 2011; Shapiro, Astin, Bishop, & Cordova, 2005). Previous studies have suggested that among healthcare staff, even short-term exposure to the distress and pain of patients can cause elevated blood pressure, anxiety, and depressive symptoms (Figley, 2002; Rubino, Luksyte, Perry, & Volpone, 2009). In the long term, such responses can cause diseases of a physical, psychological, and/or behavioral nature as well as uncomfortable relationships in the workplace that staff may experience as stressful in themselves (Grant & Kinman, 2014).

Efforts to reduce the stress that healthcare staff suffer generally attempt to integrate occupational health and safety, health promotion, and psychosocial interventions, and the effectiveness of such interventions is not clear (Hall, Doran, & Pink, 2008; Shapiro et al., 2005). It has been put forward that this is because healthcare staff are often exposed to disturbing visual images in their work (Laws, 2001; Rubino et al., 2009) and that they therefore need to address these traumatic sensual stimuli using sensual methods. It has been shown that taking part in pleasurable arts-based activities within a group context has the potential to positively influence people on physiological, biological, and social levels (Grape Viding et al., 2015). The arts have been shown to help identify a rich field of emotions and to enhance overall well-being, physically and emotionally, both individually and within groups (Simon & Graham, 2005; Warren, 2008). Furthermore, brain activity increases when stimulated with various cultural modalities at the same time; for example, visual and audio stimuli offered simultaneously trigger greater brain activity in comparison to each stimulus individually. This “multimodal” effect was proven to be successful at reducing stress in a recently published study on women with burnout syndrome (Grape Viding et al., 2015). With regards to improving health in general, cultural activities have been shown to be most effective as a preventative strategy as opposed to post-diagnosis of a condition or an illness (Bojner Horwitz, Theorell, & Anderberg, 2003; Jones, 2005; Kaye & Bleep, 1997; Malchiodi, 1999).

Engagement in the arts has been shown to not only influence at the individual, but also at the social level, improving social communication and bonding (Clift & Morrison, 2011; Davidson & Emberly, 2012; Murcia & Kreutz, 2012). Le Doux’s (1998) emotional brain theory helps to explain these effects. Arts-based activities that involve sensory experiences facilitate effects on the brain that take place without being registered cognitively. Sensory experiences “sneak through the back door into the brain” without first passing through the brain structures involved with rational or cognitive processes (such as the prefrontal cortex): Le Doux (1998) calls this the “surprise effect” of cultural activities. Le Doux argues that some stimuli travel on a “fast track” directly from the thalamus down to the amygdala nuclei—those areas of the brain associated with emotional response—without first passing through parts of the brain associated with rational thought. We become affected by stimuli without engaging in the rational process involved in understanding why.

Arts interventions have been shown to have what we call here a ripple effect, meaning that not only are patients positively affected, but also healthcare staff such as nurses, thanks to the improved atmosphere of the workplace. One study showed that healthcare staff at a medical clinic improved their physical and social well-being, enjoying a renewed sense of vitality after engaging in cultural activities in the workplace (Bygren et al., 2009). In another study, a reduction of subjective units of distress was found in healthcare staff after drawing images of stress and then transforming them into images of the experience of coping (Huss & Sarid, 2014). A large-scale longitudinal cohort study conducted on the working population, the Swedish Longitudinal Survey of Health, showed that workplaces that provide access to cultural activities during working hours help to protect their staff against burnout (Theorell et al., 2013).

The above examples serve to illustrate a developing body of literature on arts in health that shows the positive effects stemming from use of the arts in healthcare contexts (Jones, 2005; Kaye & Bleep, 1997). However, most of these studies: (1) focus on improvements for patients alone and (2) tend to focus on art as a form of self-expression rather than a participative program of engagement. In response to these limitations, this study firstly takes what we refer to as an “ecological” approach to the study of arts in healthcare. This method takes a more holistic view of the effects that engagement with the arts can have within an organization, accounting for effects on both patients and staff. The research question that guides this study therefore concerns the *overall* impact of arts-based activities. Secondly, it examines the effects of art-based activities as recreational activities that have a focus on fun rather than activities that are designed to express distress and that have a focus on stress.

The present study explores the self-reported impact of arts-based activities on healthcare providers after having participated in a range of self-chosen activities, termed a “cultural palette”, in the workplace. The purpose of the study is to provide a description of the healthcare staff’s experiences of the intervention—cultural activities within the work place. A phenomenological-hermeneutic method was chosen to capture the self-reported experiences of the participants.

## Methods

### Field of research

This research project uses the “cultural palette” (CP) model, originally developed for patients with burnout symptoms in Sweden during 2012–2014, and applies it to the stress suffered by healthcare staff (Grape Viding et al., 2015). The original CP involved engagement with different cultural activities such as music, dance, theatre, art, film, and mindfulness and was offered once a week for a 12-week period, each session lasting for 90 min (Grape Viding et al., 2015). The participants of the present study participated in a similar program of activities, which took place over a period of 10 weeks, once a week for an hour. The programs were offered at three different primary healthcare settings in Sweden. The programs of activities were developed in collaboration with healthcare staff who were asked what types of cultural activity they would want to participate in. Staff were asked to write down activities that they found enjoyable onpieces of paper, which were posted on one wall of the wards in which they worked. Those activities that were most frequently mentioned were selected for inclusion in the program. One of the healthcare centres or “units” decided on a Qi-gong activity because this was a wish from the majority of the staff members.

The cultural palette of each of the three units participating in this research was composed of the following activities:

- Unit 1/CP1: Qi-gong
- Unit 2/CP2: Yoga, line-dancing, and baking
- Unit 3/CP3: Painting, yoga, fruit and vegetable sculpting, line-dancing, and cheese and chocolate tasting

#### Recruitment

We recruited participants through a network organized by Jakobsberg’s Academic primary care center, located northwest of Stockholm. From this network, 35 primary care units were invited, and three units agreed to participate. In total, 93 members of healthcare staff took part.

### Description of cultural activities in each program

Each unit participated in a different “palette” of cultural activities, see Table 1.

**Table 1.** Activities and number of sessions by unit.

Activities	Unit 1 (*n* = 29)	Unit 2 (*n* = 38)	Unit 3 (*n* = 26)
Qi-gong	10		
Yoga		3	3
Line dancing		4	3
Baking		4	
Painting with non-dominant hand			2
Chocolate tasting			1
Cheese tasting			1
Creating sculptures from fruit and vegetables			2

### Participation rate

The proportion of staff from each ward who chose to participate in the cultural activities varied slightly: for Unit 1 the participation rate was 61%; for Unit 2 it was 40%; and for Unit 3 the rate was 56%.

## Data collection and analysis

Three focus-group interviews were conducted, one at each the healthcare center, with both the participating staff and the cultural activity leaders. All focus-group interviews were recorded, transcribed, and analysed thematically (Bojner Horwitz et al., 2003; Lindseth & Norberg, 2004; Morse & Field, 1995).

The analytical prism used for the focus-group interviews was informed by the phenomenological-hermeneutic method of the French philosopher Paul Ricouer (1976). According to this approach, analysis takes place at three levels. The first level is “Naïve Reading”, which involves a broad-brush analysis of the data that captures the researchers’ understandings of the research context from previous experience and knowledge. To conduct the analysis at this level, we read and annotated the transcript of each focus-group interview and wrote a short summary-interpretation based on our previous understandings of the context. In this case, that meant the authors’ prior experience of working with the arts in healthcare settings (Table 2).

**Table 2.** Naïve Reading.

How do the participants experience a cultural activity in the work place?
Culture is relaxing—one becomes less stressed and laugh together.
Get a new coherence with the whole working group.
New experience to see many work colleges take more space than one has seen before.
Once a week was the appropriate interval—otherwise they felt pressure and stress in their schedule.
In order for an activity to function, the management must participate with their will.
It is important that the activity is voluntary.
Good with the mixture of different cultural activities—it stimulates multiple senses.
Many perceived a slower tempo immediately after the activities.
It removes titles—does not matter what type of employment you have—everybody is equal during the cultural activities.
Feels like an individual and not like a labelled professional.
Creates a better atmosphere among colleagues.
Everybody wants to continue but no one has the strength or wants to organize it.
A wish is that cultural activities should be offered to all healthcare staff and anyone who works with people.
It becomes a breathing space in a stressful work environment.
One have received a new experience for oneself and once body in a larger context.
Become more awake after work after the day with activity.
Would have given health effects if this (CP) was an established routine.
Increased team spirit for the whole work group.
Good with an activity that is not carried out on the expense of something else.
Staff needs to get away from time to time as it is an important job.
There is a curiosity around culture being used in new ways across the complete healthcare society.
There is a sensation that financial limitations govern the whole organization.
All professionals need to add something else to their work day.
There is a feeling that is it only doctors and management that are important.
Every team member wants to feel worthy.

The second level is that of the “Structure Analyses”, which involves a closer reading of the data. We read all three interviews several times to distinguish different “meaning units”. A meaning unit is a pertinent piece of information that is relevant to the research question. The content of each meaning unit was extracted and organized into themes. The three main themes that emerged from this process of analyzing the data at this level were: (1) the physiological impact of arts-based activities, (2) arts-based activities as enhancing relationships in the workplace, and (3) challenges in implementing arts-based activities in the healthcare system.

The results of these processes constitute the basis for the third level of analysis—the “Complete Interpretation”. In the Complete Interpretation, the researchers incorporate the Naïve Reading and the result of the Structure Analyses to form an overall interpretation of the data that responds to the research question (Hubberman & Miles, 2002; Lindseth & Norberg, 2004; Ricoeur, 1976). Because the process of analysis requires these two steps, the Complete Interpretation is the part that is presented in this paper. This presentation presents a condensed synthesis of the Naïve Reading and the themes resulting from the more detailed analysis of the data itself. Quotations are provided as examples for how these themes were derived.

### Ethical considerations

All participants gave their written consent to participate in the study. Those who agreed to participate in the focus-group interviews were informed that everything they said would be coded and presented anonymously (ethical approval no. 2012/359).

### Validity

As a qualitative, interpretive study this research cannot draw on the notion of validity in the same sense as quantitative, positivist ones since the small sample and interpretive nature of the analysis produces findings that elucidate the subjective meanings that participants attribute to their experience of the cultural activities under examination (Bojner Horwitz et al., 2003; Denzin & Lincoln, 2000). These findings provide insight into the complex effects of arts-based activities as used in a healthcare setting, but are not replicable in a traditional sense. Instead, the findings inform our understanding of the multiplicity of positive effects (and therefore value) that the arts can have in organizations as an eco-system of people.

With respect to how the phenomenological-hermeneutic method was implemented, it is important to the validity of the research that the investigators have a good knowledge of the research context in order that the inclusion of their prior understandings of context in the Naïve Reading are of value. In this regard, two authors have expertise in the CP model and in arts-based interventions in Swedish healthcare settings, while another author has research experience in the use of the arts in healthcare and expertise in the problem of social worker burn-out.

### Limitations

One limitation of the data is that only three cultural settings were examined. However, the purpose of the study was to explore the experiences of participants in-depth and to examine the effects of arts-based activities on organizations in a holistic, “ecological” sense. The smaller dataset facilitated this. Another limitation is that focus groups were conducted in the presence of the cultural activity leaders. This may have made it difficult for those who had participated in them to express dissatisfaction with the experience of activities. Furthermore, there were various managers present, which may have inhibited the voices of staff members. The potential effect of having the managers present may have been lessened by the fact that the focus of the discussion was not the workplace, but the externally-provided activities (Denzin & Lincoln, 2000).

## Findings

### Theme 1: the physiological impact of arts-based activities

This theme shows how participants experienced the impact of the arts-based activities on their individual bodies, minds, and emotions. This was characterized by two physical experiences: (1) feelings of joy and stimulation from learning a new skill and (2) feelings of relaxation and “slowing down”, with some suggestion that these effects could be sustained after the activities had ended. The following quotes illustrate these two experiences:

[I] quickly wind down and achieve an inner calmness.

A feeling of both desire and joy.

The brain disconnects from all problems.

Reduced blood pressure.

[It] becomes a breathing space in a stressful work environment.

I have created new memories that I can return to afterwards.

[It has a] positive effect on me as a person.

[I feel] satisfaction by learning something, one effect is enrichment—learning something new.

It was relaxing and enriching.

[I] became happy, walked away happy, and when I think of it I become happy.

The experience of positive social interaction during participation of the CP activities was characterized by a sense of group coherence and pleasurable interactions among participants. Outside of the activities it seemed that the unit members talked about the activities they had experienced, which created a sense of community through shared experience and shared emotions. The laughter of joy and feelings of trust created between members during the activities continued beyond, into the day-to-day of the workplace.

### Theme 2: arts-based activities as enhancing relationships in the workplace

Another finding was that it seems that the arts-based activities somewhat challenged the formal organizational hierarchies between members of staff and helped to transform the culture of the workplace: staff members became recognized as individuals to a greater extent, so participants felt less obligated to perform what was perceived to be an artificial identity in front of colleagues. Reinforcing this, it was reported that there was greater interaction among different staff levels. The following quotes show examples of this theme:

[It’s] nice to do something together with colleagues.

Gave [me] a sense of coherence with the whole group.

Glad to see so many people participating more actively in the group than you normally see.

We walked slower after the activity, almost like flying, not at all the ordinary “hunting” mode we work in.

[I] became an individual and not a labelled professional.

Takes away working roles, does not matter where you stand on the ladder. Everyone becomes equal.

No need to perform, just be oneself without obligation.

Everybody becomes equal when you start to see beyond one’s profession.

The work climate has improved and a more respectful environment is established among the co-workers.

We did not laugh at each other, but laughed together.

The experience of laughing about doing it “wrong” with co-workers was important.

Go and see a play, we can do that too, but then it becomes something else; what is important is that we do the cultural activities together.

This theme pointed to the importance of the arts-based activities for the participants, together with the difficulties they experienced in finding time to participate in them. Participants showed a recognition of how much of a challenge it is to find space for such interventions in the healthcare workplace.

### Theme 3: challenges in implementing arts-based activities in the context of the healthcare system

This was true for staff at all hierarchical levels. Finding time was a factor, alongside the stress involved in setting up the program and maintaining motivation to run it:

[It was] important that the transportation did not take too much time if it takes place during work hours.

Space must be made by the organization and by society at large.

Everybody wants to participate but no one wants to organize it.

In order for an activity to work, the management must participate.

It can be dangerous to have activities as compulsory.

Time is needed to get back to work after the activity.

To “be allowed” as an employee of a public institution to enjoy cultural activities during work hours is a challenge, and at the same time it feels like a luxury.

Society will earn from this in the long term.

## Discussion

In this study, we sought to investigate the effects of an art-based intervention in a healthcare setting in a more holistic way than in existing literature. Rather than focus on the positive outcomes for patients of self-expression through art, we examined how staff participation in arts-based activities could help to alleviate stress by taking advantage of how sensory engagement in the arts can “fast track” effects on the brain through emotion, circumventing cognitive processes (Le Doux, 1998). We took an “ecological” approach to the data, looking for ways in which engagement in the activities had positive effects beyond the individual—to the social and organizational.

We found that the activities of the CP facilitated three themes of experience for healthcare staff: the first theme describes the ways in which the cultural activities were perceived to bring health and emotional benefits to the individual; within the second theme, the benefits of the activities were at the level of the social, permitting a shift in the dynamic of interaction between people in the work place; finally the third theme speaks of the challenges of the implementation of the arts-based activities within the context of healthcare.

In terms of individual embodied experiences, participation in arts-based activities enabled healthcare staff to relax and to enjoy themselves, which was associated with improved health through a sense of slowing down, of letting go, and of being in the moment (“Quickly wind down and achieve an inner calmness”) and though joy and a feeling of vitality (“A feeling of both desire and joy”). This finding is consistent with other research on arts and health that shows the ability of the arts to reduce stress, enhance mood, and enhance connection between left and right brain functions, creating new neurological pathways between emotional and cognitive areas of the brain (Baumgartner, Lutz, Schmidt, & Jäncke, 2006; Clift & Hancox, 2001; Cohen et al., 2006; Duberg, Möller, & Sunvisson, 2016; Immordino-Yang, McColl, Damasio, & Damasio, 2009; Le Doux, 1988). It is thought that the multimodality of arts-based activities improves this effect (Bell & Robbins, 2007; De Petrillo & Winner, 2005; Hass-Cohen, 2003; Pennebaker, 1997). The present study contributes to this discussion by showing that even engagement with arts-based activities that are not specifically designed to reduce stress, such as sculpting chocolate or line-dancing, also generate these positive effects. Building on the findings of existing literature that center on improving patient health, for staff, self-expression was not a necessary feature of those activities that produced therapeutic effects. The integration of relaxation, pleasure, and learning may exemplify Csikszentmihalyi’s (1990) description of a state of “flow”—an alert, mindful state of concentration—through interaction with the arts. This goes beyond current suggestions in the literature that the arts are most helpful for hospital workers because both secondary trauma—the distress caused by witnessing the traumatic experiences of others—and participation in artistic activities both involve sensory experience (Hass-Cohen, 2003).

The second theme showed how the shared experience of the arts-based activities transformed the social interaction among the healthcare staff. Two processes can be delineated within this transformation. First, the arts interaction created an opportunity to get to know fellow workers as individuals (“[I] became an individual and not a labelled professional”). Second, the interaction involved in the arts-based activities broke down the formal hierarchical relations of the medical world; at this level, the arts served to humanized the working environment wherein colleagues could appreciate each other as subjects rather than objects of their roles. Through engaging in physical and non-outcome focused group activities, emotions and behaviors were brought into the workplace that would normally have been seen to belong outside of it. This shifted the dynamic of staff interactions and changed the atmosphere of the workplace (“many people participating more actively in the group then they normally see”). This helped to reduce the hierarchy and differentiation by work role and enabled everyone to portray themselves beyond their professional identity and to become recognized as a “whole” person. This finding likely stems from the communicative aspects of engaging in creative processes together. The group arts-based activities serve as a non-judgmental context as described Merns and Thomas (2007) and Rogers (1995). The focus of the activity is not on any product, but on the process and on interaction itself (“No need to perform, just be yourself without obligation”).

To perform effectively, healthcare staff require a degree of formal structure to fulfil their results-oriented roles: this may influence the culture of the workplace. When this becomes too mechanistic and rigid, then both healthcare staff and patients can suffer (Huss, 2012). We see that arts-based activities enable a shift in this, without directly challenging the chains of command and relations of responsibility that are needed in an efficient healthcare system. The activities thus go beyond “fun” to offer a transformation of healthcare work culture. This connects to the role of arts in creating social change. The transformative role of art is well documented in the literature (Butler, 2001; Foster, 2007; Huss, 2012).

Regarding the third theme, the struggles that participants talked about in finding a way to set up and to run a program of arts-based activities within the healthcare setting may be indicative of the challenge that they made to the existing hierarchy, work focus, and results-oriented culture (Jones, 2005; Kaye & Bleep, 1997). A psychological explanation for the difficulties in implementing a relaxing and playful activity could be the difficulty in breaking the habituated patterns of behavior that are associated with stress. Moreover, stress may be caused not only by the nature of the healthcare setting itself in which patients are in distress, but also by the reduction in public funds that can result in overwork (Grant & Kinman, 2014).

Overall, we see that the arts-based activities introduced to healthcare staff were potentially transformative of individual social interaction and culture. Whilst this qualitative, phenomenological-hermeneutic study has limitations with regards to generalizability, it offers a mapping out of different possible effects of arts-based intervention in a healthcare eco-system of staff and patients. The degree to which these effects are seen in different types of healthcare organization can be tested with broader studies.

The theoretical implications of the study concern the beneficial effect of these recreational arts-based activities for healthcare staff and the healthcare organization and the fact that because the activities were regarded not as work but as something additional to work, or even “play”, there was some difficulty in creating space for them with regards to time and energy. Despite the perception that the activities do not constitute work, it is important to note that these types of activities are much cheaper and more accessible then other stress-reduction interventions. Moreover, they potentially have a positive impact at three levels: individual, group, and workplace. These three levels create an ecological map of effects that go beyond the current literature on the ability of art to calm and enhance the mood of an individual. It may be argued that these transformations, which help to care for the staff, are also a step towards providing better care for patients.

The inclusion of cultural activities for healthcare staff may offer a cheap and therefore sustainable way to receive these multiple benefits. Cultural activities may therefore be valuable in planning strategically for the prevention of stress and in enabling the creation of sustainable healthcare systems (Missimer, Robèrt, Broman, & Sverdrup, 2010).
